# Characterization of a Novel Megalocytivirus Isolated from European Chub (*Squalius cephalus*)

**DOI:** 10.3390/v11050440

**Published:** 2019-05-15

**Authors:** Maya A. Halaly, Kuttichantran Subramaniam, Samantha A. Koda, Vsevolod L. Popov, David Stone, Keith Way, Thomas B. Waltzek

**Affiliations:** 1 Department of Animal Sciences, College of Agricultural and Life Sciences, University of Florida, Gainesville, FL 32611, USA; mayah123@ufl.edu; 2 Department of Infectious Diseases and Immunology, College of Veterinary Medicine, University of Florida, Gainesville, FL 32611, USA; kuttichantran@ufl.edu (K.S.); samanthakoda@ufl.edu (S.A.K.); 3 Department of Pathology, University of Texas Medical Branch, Galveston, TX 77555, USA; vpopov@utmb.edu; 4 Centre for Environment, Fisheries and Aquaculture Science (CEFAS), Weymouth DT4 8UB, UK; david.stone@cefas.co.uk (D.S.); keith.way@cefas.co.uk (K.W.)

**Keywords:** megalocytivirus, iridovirus, European chub

## Abstract

A novel virus from moribund European chub (*Squalius cephalus*) was isolated on *epithelioma papulosum cyprini* (EPC) cells. Transmission electron microscopic examination revealed abundant non-enveloped, hexagonal virus particles in the cytoplasm of infected EPC cells consistent with an iridovirus. Illumina MiSeq sequence data enabled the assembly and annotation of the full genome (128,216 bp encoding 108 open reading frames) of the suspected iridovirus. Maximum Likelihood phylogenetic analyses based on 25 iridovirus core genes supported the European chub iridovirus (ECIV) as being the sister species to the recently-discovered scale drop disease virus (SDDV), which together form the most basal megalocytivirus clade. Genetic analyses of the ECIV major capsid protein and ATPase genes revealed the greatest nucleotide identity to members of the genus *Megalocytivirus* including SDDV. These data support ECIV as a novel member within the genus *Megalocytivirus*. Experimental challenge studies are needed to fulfill River’s postulates and determine whether ECIV induces the pathognomonic microscopic lesions (i.e., megalocytes with basophilic cytoplasmic inclusions) observed in megalocytivirus infections.

## 1. Introduction

The European chub (*Squalius cephalus*) is a rheophilic cyprinid that is widely distributed throughout Eurasia [1,2]. Although they are omnivores, adults include a greater portion of fish in their diets [3,4]. They are popular among amateur anglers because they readily take a variety of live and artificial baits and reach a maximum reported standard length of 60 cm [4]. Ireland and Italy have raised concerns that the recent introductions of European chub may threaten their native biodiversity [1,2].

Cyprinid fishes are susceptible to a range of RNA and DNA viruses (reviewed in [5]). Iridoviruses have been described in a wide variety of fishes; however, few iridoviruses have been described from cyprinids [6,7]. Although an irido-like virus was isolated from the gills and kidneys of moribund common carp (*Cyprinus carpio*; i.e., common carp iridovirus; CCIV), the role of CCIV in disease could not be established (reviewed in [5,8]). In addition, two irido-like viruses were isolated from the swim bladder of healthy goldfish (*Carassius auratus),* goldfish virus-1 and goldfish virus-2, have been proposed as members of the family *Iridoviridae* based on biophysical and biochemical analyses [9]. Recent studies have reported the detection of the iridovirus, infectious spleen and kidney necrosis virus (ISKNV), in goldfish and common carp traded in Brazil [10] and in zebrafish (*Danio rerio*) from a research facility in Spain [11]. In addition, a Santee-Cooper ranavirus strain was isolated from diseased koi carp and shown experimentally to induce lethal disease [12].

Members of the family *Iridoviridae* possess large nucleocapsids that display icosahedral symmetry (120–200 nm in diameter) and encapsidate a linear, double-stranded DNA genome. The family is divided into two subfamilies, *Alphairidovirinae* and *Betairidovirinae* [13]. The former consists of three genera (*Ranavirus*, *Lymphocystivirus*, and *Megalocytivirus*) that are known to infect ectothermic vertebrates including fish, amphibians, and reptiles [13,14]. Megalocytiviruses are globally emerging viruses, causing lethal systemic infections in wild and cultured freshwater, brackish, and marine fishes [15]. Recent megalocytivirus phylogenetic analyses based on the major capsid protein (MCP) and ATPase genes revealed that the species, ISKNV, is divided into three genotypes including (1) ISKNV, which was first described from farmed mandarin fish (*Siniperca chuatsi*) reared for food in China [16,17]; (2) red sea bream iridovirus (RSIV), which was initially reported in cultured red sea bream (*Pagrus major*) from Japan [18]; and (3) turbot reddish body iridovirus (TRBIV), characterized from flatfishes (order Pleuronectiformes) reared for food in the Yellow Sea in East Asia [19]. In 2012, the threespine stickleback iridovirus (TSIV) was characterized from wild-caught threespine stickleback (*Gasterosteus aculeatus*) from Canada, and based on genetic and phylogenetic analyses, it was proposed as a novel megalocytivirus species [20]. Recently, an epizootic involving Asian seabass (*Lates calcarifer*) cultured in Singapore was found to be caused by a divergent megalocytivirus, scale drop disease virus (SDDV; [21]).

In this investigation, we described the *in vitro* growth characteristics, ultrastructural pathology, and phylogenomic characterization of the first iridovirus isolated from European chub. The cytopathic effect (i.e., enlarged, rounded, refractile cells) induced by this virus and its ultrastructural features are consistent with that of a megalocytivirus. The genetic and phylogenetic analyses further supported this virus as a divergent megalocytivirus, referred to as European chub iridovirus.

## 2. Materials and Methods

### 2.1. Case History

A hatchery located in the English Midlands of the UK, rearing cyprinids for wild stock enhancement, experienced increased mortality in several species, including European chub. Live European chub juveniles were transported to the Center for Environmental Fisheries and Aquaculture Science (CEFAS), in Weymouth, England and were subsequently euthanized after they appeared moribund upon arrival. Internal tissue homogenates were inoculated onto the following cell lines using standard virological methods: bluegill fry (BF-2), *epithelioma papulosum cyprini* (EPC), chinook salmon embryo (CHSE-214), koi fin (KF-1), and common carp brain (CCB). Cytopathic effects (CPE) were observed as early as 48 h post-inoculation, resulting in the appearance of enlarged refractile cells in all cell lines at 20 °C. 

### 2.2. Cell Culture

The European chub iridovirus (ECIV) isolate grown on EPC cells was sent from CEFAS to the Wildlife and Aquatic Veterinary Disease Laboratory in Gainesville, Florida, USA. The virus isolate was then inoculated onto confluent monolayers of EPC cells maintained in MEM media with 10% fetal bovine serum and 1% HEPES (4-(2-hydroxyethyl)-1-piperazineethanesulfonic acid) at 25 °C and monitored daily for CPE. Flasks of EPC cells displaying CPE were used to characterize the ultrastructural and genetic properties of ECIV.

### 2.3. Transmission Electron Microscopy

The ECIV isolate was propagated in a 75 cm^2^ flask of EPC cells until CPE was observed. The supernatant from the infected flask was discarded, and the monolayer was fixed in 15 mL of modified Karnovsky’s fixative (2P + 2G, 2% formaldehyde prepared from paraformaldehyde and 2% glutaraldehyde in 0.1 M cacodylate buffer pH 7.4) at room temperature for 1 h. The monolayer was then washed in cacodylate buffer, scraped off the flask, and pelleted. The pellet was shipped via PBS overnight on ice packs to the University of Texas Medical Branch Department of Pathology Electron Microscopy Laboratory (UTMB-EML). At UTMB-EML, the cell pellet was washed in cacodylate buffer and left in 2P + 2G fixative overnight at 4 °C. The next day, the cell pellet was washed twice in cacodylate buffer, post-fixed in 1% OsO_4_ in 0.1 M cacodylate buffer pH 7.4, en bloc stained with 2% aqueous uranyl acetate, dehydrated in ascending concentrations of ethanol, processed through propylene oxide, and embedded in Poly/Bed 812 epoxy plastic (Polysciences, Warrington, PA, USA). Ultrathin sections were cut on a Leica ULTRACUT EM UC7 ultramicrotome (Leica Microsystems, Buffalo Grove, IL, USA), stained with 0.4% lead citrate, and examined in a JEM-1400 electron microscope (JEOL USA) at 80 kV. 

### 2.4. DNA Extraction, Whole Genome Sequencing, and Assembly

Inoculation of the ECIV isolate onto EPC cells in four 175 cm^2^ flasks at a high multiplicity of infection provided third-passage material harvested after 21 days post-infection when CPE was extensive. Cell culture supernatant was clarified at 5520× *g* for 20 min at 4 °C. The pelleted virus was obtained by centrifugation of the clarified supernatant at 100,000× *g* for 1.25 h at 4 °C. The viral pellet was resuspended in 360 μL of animal tissue lysis (ATL) buffer prior to extraction of viral genomic DNA using a DNeasy Blood and Tissue Kit (Qiagen, Germantown, MD, USA) according to the manufacturer’s instructions. A DNA library was generated using a Nextera XT DNA Kit, and sequencing was performed using a V3 chemistry 600 cycle Kit on a MiSeq sequencer (Illumina, Germantown, MD, USA). *De novo* assembly of the paired-end reads was performed in SPAdes 3.5.0 genome assembly algorithm [22]. The quality of the genome assembly was verified by mapping the reads back to the consensus sequence in Bowtie 2 2.1.0 [23] and visually inspecting the alignment in Tablet 1.14.10.20 [24].

### 2.5. Genome Annotation, Genetic, and Phylogenetic Analysis

The viral genome was annotated using GenemarkS [25], and the functions were predicted based on BLASTP searches against the National Center for Biotechnology Information (NCBI) GenBank non-redundant (nr) protein sequence database and conserved domain database. A total of 25 iridovirus core genes were used to conduct the Maximum Likelihood (ML) analysis for 47 iridoviruses, including ECIV (Table 1). The amino acid (AA) sequence alignments were performed for each gene in MAFFT 5.8 using default parameters [26] and concatenated using Geneious R10 [27]. The final dataset contained 19,340 AA characters, and the phylogenetic tree was constructed using IQ-Tree [28] with default parameters. In addition, genetic analyses were performed using the Sequence Demarcation Tool v1.2 with the MAFFT alignment option implemented [29] to compare the nucleotide sequence identity of megalocytiviruses based on the major capsid protein and ATPase gene alignments. 

## 3. Results

### 3.1. Cell Culture

**Figure 1:**
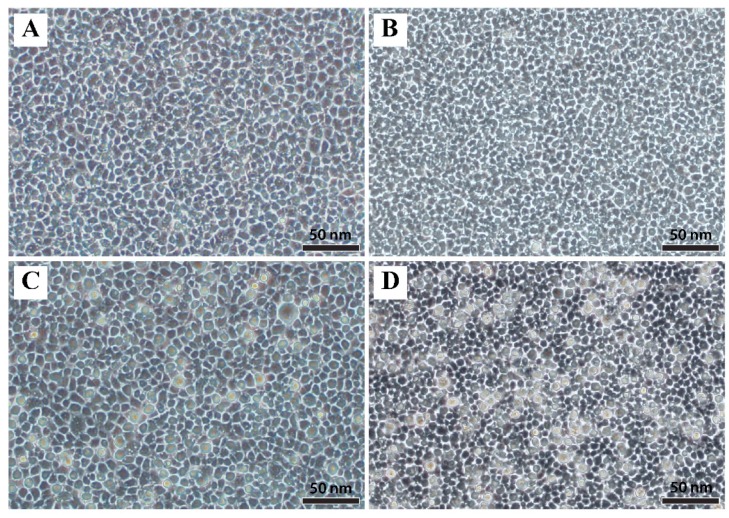
Microscopic examination of *epithelioma papulosum cyprini* cells infected with European chub iridovirus. (**A**) Control flask at 48 h post-inoculation (hpi); (**B**) control flask 96 hpi; (**C**) infected flask showing enlarged and refractile cells at 48 hpi; (**D**) infected flask showing enlarged and refractile cells at 96 hpi. Scale bars are 50 µm.

The CPE in the EPC cell line consisted of enlarged refractile cells observed within 48 h post-inoculation (hpi). Extensive CPE of the monolayer was observed by 96 hpi, at which point, clumps of affected cells were observed and began to detach from the monolayer (Figure 1).

### 3.2. Transmission Electron Microscopy

**Figure 2:**
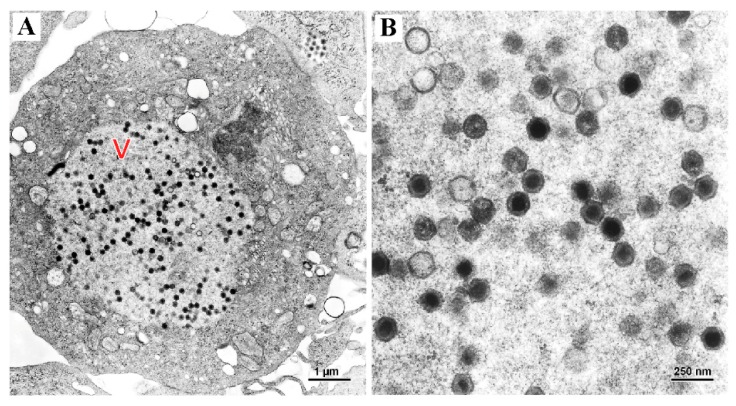
(**A**) Transmission electron photomicrograph of an *epithelioma papulosum cyprini* cell infected with European chub iridovirus, displaying numerous non-enveloped, hexagonal viral particles within the viral assembly site (labeled as V) in the cytoplasm. Scale bar is 1 µm. (**B**) Higher magnification of the virus particles. Scale bar is 250 nm.

Non-enveloped, hexagonal virus particles with electron-lucent or electron-dense cores were observed within viral assembly sites in the cytoplasm of infected EPC cells (Figure 2). The mean diameter of individual virus particles was 127 nm from opposite sides (*n* = 20, standard deviation = 9) and 147 nm from apex to apex (*n* = 20, standard deviation = 10).

### 3.3. Genome Annotation, Genetic and Phylogenetic Analyses

The *de novo* assembly of the 14,420,600 paired-end reads recovered a contiguous sequence of 128,216 bp with an overall coverage of 2948 reads/nucleotide. The %GC of the genome was 38.83, and a total of 108 open reading frames (ORFs) were predicted (Table S1). Comparative genomic analysis revealed the absence of one iridovirus core gene (ISKNV ORF 32R encoding putative thymidine kinase, GenBank accession number AF371960; SDDV ORF 125L, GenBank accession number KR139659) in Europoean chub iridovirus (ECIV). Eighty-seven genes showed the highest amino acid (AA) sequence identity to SDDV, seven genes to various members of the family *Iridoviridae*, and six genes to other organisms (e.g., eukaryotes including fish and fungi). Eight genes were found to be unique to the ECIV genome and did not display similarity to existing genes within the NCBI GenBank nr protein sequence database. Of these, seven genes (ORFs 2, 5, 18, 58, 65, 82, and 89) were predicted as hypothetical proteins, and ORF 66 predicted to be a chromosome segregation protein (Table S1). As with cherax quadricarinatus iridovirus strain CQIV-CN01 (GenBank acc. MF197913), shrimp hemocyte iridescent virus isolate 20141215 (GenBank accession number MF599468), grouper iridovirus (GenBank accession number AY666015), and Singapore grouper iridovirus (GenBank acc. no. AY521625), the ECIV ORF 84 is predicted to encode an ubiquitin family protein. The ECIV ORFs 31 and 57 were predicted as members of the serpin superfamily and showed the highest AA to SDDV ORFs 97L and 45R, respectively. The ECIV encoded a HIRAN domain containing protein (ORF 49) and a family of ankyrin (ANK) proteins (ORFs 3, 4, 44, 46, 47, 63, 92, 99, and 102) that ranged in size from 143 to 478 AA residues. Each ECIV ANK protein possesses between 1 and 7 ANK motifs. ECIV ORF 97 was predicted to encode an US22 protein and displayed the highest AA sequence identity (39.2%) to an US22 protein from Asian swamp eel (*Monopterus albus*). The complete genome sequence of ECIV has been deposited in NCBI GenBank under the accession number MK637631. 

**Figure 3:**
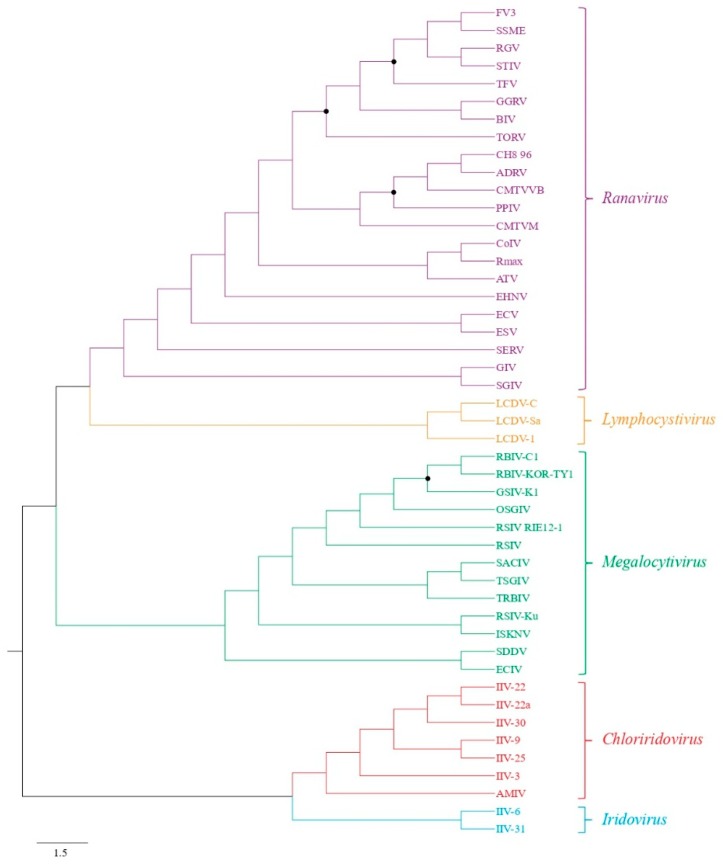
Cladogram depicting the relationship of European chub iridovirus to 47 other members of the family *Iridoviridae* based on 25 core genes. The Maximum Likelihood tree was generated using 1000 bootstraps and the branch lengths are based on the number of inferred substitutions, as indicated by the scale. All nodes were supported by bootstrap values >80% except those labeled with black circles. See Table 1 for virus abbreviations.

The ML analysis of the concatenated 25 iridovirus core gene sequences produced a well-resolved and supported tree (Figure 3). The ECIV was found to be the sister species to the SDDV, which together form the basal branch of the megalocytivirus tree. Genetic comparisons of the ECIV ATPase and MCP nucleotide sequences to other megalocytiviruses ranged from 66.4 to 76.9% and 62.8 to 73.1%, respectively (Tables S2 and S3). The highest identities were observed between ECIV and SDDV.

## 4. Discussion

In this investigation, we report the complete genome sequence of a novel iridovirus isolated from moribund European chub (*Squalius cephalus*) in England. The *in vitro* characteristics (i.e., enlarged, rounded, and refractile cells), virion ultrastructure and morphogenesis (i.e., non-enveloped hexagonal virus particles within the cytoplasm), and genetic/phylogenetic analyses supported the identification of European chub iridovirus (ECIV) as a divergent megalocytivirus most closely related to the recently described SDDV [21]. To our knowledge, this study represents the first isolation and genomic characterization of a megalocytivirus in a cyprinid fish. The results of these studies add to a growing body of literature on the host range and threat megalocytiviruses pose to wild and cultured fishes, including their potential impacts in cyprinids [7].

The type species of the genus *Megalocytivirus* (*Infectious spleen and kidney necrosis virus*; ISKNV) exhibits low host specificity, with strains infecting >150 species of freshwater, brackish, and marine fishes [7,30]. The recent characterization of the TSIV from Canadian threespine stickleback (*Gasterosteus aculeatus*) [20] and SDDV from Asian seabass (*Lates calcarifer*) [21] have revealed the existence of genetically-divergent megalocytiviruses that have been argued to represent new species. The discovery of iridoviruses distantly related to ISKNV has stimulated discussion by members of the International Committee on Taxonomy of Viruses study group on iridoviruses to begin re-evaluating the criteria used in defining megalocytivirus species [13]. The genome annotation of ECIV revealed that it possesses 108 predicted genes (including eight unique genes), and compared to other fully-sequenced megalocytivirus genomes, ECIV has the longest genome and a low %GC content similar to SDDV. These data, taken together with the genetic and phylogenetic analyses, suggest ECIV represents yet another novel megalocytivirus, and we propose the formal species designation of European chub iridovirus to be considered for approval by the International Committee on Taxonomy of Viruses.

The HIRAN domain-containing protein (ECIV ORF 49) is not observed in other viruses, except in some bacteriophages [31], and displayed the highest amino acid (AA) sequence identity (39.1%) to the protein of a zygomycete fungus (*Basidiobolus meristosporus*). The HIRAN domain has been found as a standalone protein in a wide range of bacteria or fused to other catalytic domains in eukaryotes [31]. The HIRAN domain is predicted to function as a DNA-binding domain that recognizes damaged DNA or stalled replication forks and recruits repair and remodeling enzymes to these sites [31]. Although a variety of DNA viruses encode serpin proteins, lymphocystis disease virus Sa isolate SA9 (ORF 50R; GenBank accession number KX643370), SDDV (ORFs 45R and 97L; GenBank accession number KR139659), and ECIV (ORFs 31 and 57) are the only iridoviruses that possess these genes [32]. Recent studies have demonstrated that poxvirus-encoded serpins subvert host immune responses by inhibiting the inflammatory response and apoptosis [33]. Megalocytiviruses are the only member of the family *Iridoviridae* to encode ANK repeat proteins, and ECIV encodes the greatest number of copies (ORFs 2, 5, 18, 58, 65, 82, and 89) among members of the genus. ANK repeat proteins have also been described in poxviruses, mimiviruses, and phycodnaviruses. The ISKNV ANK repeat protein (ISKNV ORF 124L; GenBank accession number AF371960) has been shown to interfere with TNF-α-induced NF-κB activation, an important immune regulatory pathway [34]. Poxvirus-encoded ANK repeat proteins are suggested to be involved with host cell tropism [35] and manipulation of the host cell ubiquitin-proteasome machinery [36]. The US22 proteins are present in all megalocytiviruses, except in SDDV and ISKNV, and these proteins are believed to counter diverse host immune responses by interacting with specific host proteins [37,38]. The highest AA sequence identity of the ECIV US22 protein (ORF 97) to Asian swamp eel suggests it was acquired from a fish host.

The *in vitro* cultivation of megalocytiviruses is challenging, with propagation reported in a handful of cell lines including the grunt fin cell line for RSIV and three spot gourami iridovirus [17,30], the mandarin fish fry cell line for ISKNV [39], and the turbot fin cell line for TRBIV [40]. Commonly-used cell lines failed in the propagation of the Banggai cardinalfish iridovirus, a strain of the ISKNV genotype, including the *epithelioma papulosum cyprini* (EPC), bluegill fry (BF-2), chinook salmon embryo (CHSE-214), and fathead minnow (FHM) cell lines [41]. Similarly, TSIV was refractory to culture on EPC, BF-2, and CHSE-214 cell lines [20]. In contrast, ECIV is less fastidious than other megalocytiviruses growing on EPC, BF-2, CHSE-214, KF-1, and CCB cell lines. Whether the related SDDV shares similar *in vitro* growth characteristics with ECIV remains to be determined as SDDV has only been tested and cultivated in the seabass kidney cell line [21].

Future challenge studies will be needed to determine whether ECIV causes disease in European chub and related cyprinids. These experiments will also help determine whether ECIV induces the pathognomonic microscopic lesions (i.e., megalocytes with basophilic cytoplasmic inclusions) observed in all other megalocytivirus infections to date [17,20,21,30]. Finally, the genomic sequence presented here will facilitate the development of molecular diagnostic assays that could be used to determine the prevalence of ECIV among European chub populations across Eurasia.

## Figures and Tables

**Table 1:** GenBank accession numbers for the full genome sequences of iridoviruses used in the 25 iridovirus core gene phylogenetic analysis.

Species Name (Virus Abbreviation)	Genus	GenBank Acc. No.
*Anopheles minimus iridovirus* (AMIV)	*Chloriridovirus*	KF938901
*Invertebrate iridovirus 22* (IIV-22)	*Chloriridovirus*	HF920633
Invertebrate iridovirus 22a (IIV-22a)	*Chloriridovirus*	HF920634
*Invertebrate iridescent virus 3* (IIV-3)	*Chloriridovirus*	DQ643392
Invertebrate iridescent virus 30 (IIV-30)	*Chloriridovirus*	HF920636
*Invertebrate iridescent virus 9* (IIV-9)	*Chloriridovirus*	GQ918152
*Invertebrate iridescent virus 25* (IIV-25)	*Chloriridovirus*	HF920635
*Invertebrate iridescent virus 31* (IIV-31)	*Iridovirus*	HF920637
*Invertebrate iridescent virus 6* (IIV-6)	*Iridovirus*	AF303741
*Lymphocystis disease virus 1* (LCDV-1)	*Lymphocystivirus*	L63545
*Lymphocystis disease virus 2* (LCDV-C)	*Lymphocystivirus*	AY380826
*Lymphocystis disease virus 3* (LCDV-Sa)	*Lymphocystivirus*	PRJEB12506
European chub iridovirus (ECIV)	*Megalocytivirus*	MK637631
Giant seaperch iridovirus (GSIV-K1)	*Megalocytivirus*	KT804738
*Infectious spleen and kidney necrosis virus* (ISKNV)	*Megalocytivirus*	AF371960
Infectious spleen and kidney necrosis virus (RSIV-Ku)	*Megalocytivirus*	KT781098
Orange-spotted grouper iridovirus (OSGIV)	*Megalocytivirus*	AY894343
Red seabream iridovirus (RSIV)	*Megalocytivirus*	AB104413
Red seabream iridovirus (RSIV RIE12–1)	*Megalocytivirus*	AP017456
Rock bream iridovirus (RBIV-KOR-TY1)	*Megalocytivirus*	AY532606
Rock bream iridovirus (RBIV-C1)	*Megalocytivirus*	KC244182
*Scale drop disease virus* (SDDV)	*Megalocytivirus*	KR139659
South American cichlid iridovirus (SACIV)	*Megalocytivirus*	MG570131
Turbot reddish body iridovirus (TRBIV)	*Megalocytivirus*	GQ273492
Three spot gourami iridovirus (TSGIV)	*Megalocytivirus*	MG570132
*Ambystoma tigrinumvirus* (ATV)	*Ranavirus*	AY150217
Andrias davidianus ranavirus (ADRV)	*Ranavirus*	KC865735
Bohle iridovirus (BIV)	*Ranavirus*	KX185156
Cod iridovirus (CoIV)	*Ranavirus*	KX574342
*Common midwife toad virus* (CMTVM)	*Ranavirus*	JQ231222
*Common midwife toad virus* (CMTVVB)	*Ranavirus*	KP056312
*Epizootic haematopoietic necrosis virus* (EHNV)	*Ranavirus*	FJ433873
European catfish virus (ECV)	*Ranavirus*	KT989885
European sheatfish virus (ESV)	*Ranavirus*	JQ724856
*Frog virus 3* (FV3)	*Ranavirus*	AY548484
Frog virus 3 isolate SSME (SSME)	*Ranavirus*	KF175144
German gecko ranavirus (GGRV)	*Ranavirus*	KP266742
Grouper iridovirus (GIV)	*Ranavirus*	AY666015
Pike perch iridovirus (PPIV)	*Ranavirus*	KX574341
Rana grylio iridovirus (RGV)	*Ranavirus*	JQ654586
Ranavirus maximus (Rmax)	*Ranavirus*	KX574434
Short-finned eel ranavirus (SERV)	*Ranavirus*	KX353311
*Singapore grouper iridovirus* (SGIV)	*Ranavirus*	AY521625
Soft-shelled turtle iridovirus (STIV)	*Ranavirus*	EU627010
Testudo hermanni ranavirus (CH8/96)	*Ranavirus*	KP266741
Tiger frog virus (TFV)	*Ranavirus*	AF389451
Tortoise ranavirus isolate (ToRV1)	*Ranavirus*	KP266743

## Supplementary Materials

The following are available online at https://www.mdpi.com/1999-4915/11/5/440/s1, Table S1: Genome annotation of the European Chub iridovirus; Table S2: Genetic relationship among iridoviruses measured as nucleotide sequence identity in the major capsid protein. Values for European chub iridovirus are outlined in red. See Table 1 for virus abbreviations; Table S3: Genetic relationship among iridoviruses measured as nucleotide sequence identity in the ATPase gene. Values for European chub iridovirus are outlined in red. See Table 1 for virus abbreviations.
