# Supplementary material for: Characterization of a Novel Megalocytivirus Isolated from European Chub (Squalius cephalus)
Source: Viruses. 2019 May 15;11(5):440. doi: 10.3390/v11050440 (PMC6563503; doi:10.3390/v11050440)
Supplement: Supplementary file 1 [file viruses-11-00440-s001.pdf]

Table S1. Genome annotation of the European chub iridovirus.

ORF	Position (nt range)	Product size (AA)	Predicted function and conserved domain or signature	Best BLASTP hit[a]		
				Description	E-value	Accession no.
1	1-1,125	374	Transmembrane amino acid transporter protein	ORF_082R [Scale drop disease virus]	4.10E-166	YP_009163843
2	1,792-2,103	103	Hypothetical protein			
3	2,247-3,512	421	Ankyrin repeat-containing protein	ORF_083L [Scale drop disease virus]	1.73E-116	YP_009163844
4	3,399-4,328	309	Ankyrin repeat-containing protein	ORF_084R [Scale drop disease virus]	1.01E-128	YP_009163845
5	4,304-4,696	130	Hypothetical protein			
6	4,774-6,267	497	mRNA capping enzyme	putative RNA guanylytransferase [Infectious spleen and kidney necrosis virus]	9.84E-145	NP_612286
7	6,280-6,909	209	Thymidylate kinase	ORF_089L [Scale drop disease virus]	3.30E-35	YP_009163850
8	7,117-7,587	156	Hypothetical protein	hypothetical protein [South American cichlid iridovirus]	7.00E-10	AVR29722
9	7,928-8635	235	Src homology 2 (SH2) domain-containing protein	ORF_117L [Scale drop disease virus]	1.86E-08	YP_009163878
10	8,987-9,760	257	Ribonuclease III	ORF_025R [Scale drop disease virus]	4.54E-130	YP_009163786
11	9,776-10,213	145	Hypothetical product	ORF_024R [Scale drop disease virus]	2.20E-70	YP_009163785
12	10,220-10,762	180	Hypothetical protein	ORF_023R [Scale drop disease virus]	3.92E-29	YP_009163784
13	10,858-11,235	125	Hypothetical protein	ORF_021L [Scale drop disease virus]	5.46E-26	YP_009163782

Table S1. Continued.

ORF	Position (nt range)	Product size (AA)	Predicted function and conserved domain or signature	Best BLAST hit[a]		
				Description	E-value	Accession no.
14	11,281-11,568	95	Hypothetical protein	ORF_019L [Scale drop disease virus]	3.42E-38	YP_009163780
15	11,630-12,970	446	Hypothetical protein	ORF_072R [Scale drop disease virus]	4.82E-21	YP_009163833
16	12,980-13,555	191	Short chain dehydrogenase	ORF_071R [Scale drop disease virus]	5.15E-79	YP_009163832
17	13,552-16,491	979	DNA polymerase family B	ORF_070R [Scale drop disease virus]	0	YP_009163831
18	17,113-17,412	99	Hypothetical protein			
19	17,427-18,056	209	Hypothetical protein	ORF_069L [Scale drop disease virus]	8.38E-86	YP_009163830
20	18,059-18,406	115	Hypothetical protein	ORF_119R [Scale drop disease virus]	3.48E-48	YP_009163880
21	18,440-19240	266	Hypothetical protein	ORF_118L [Scale drop disease virus]	8.77E-54	YP_009163879
22	19,304-22,435	1043	DNA-dependent RNA polymerase II second largest subunit	ORF_122L [Scale drop disease virus]	0	YP_009163883
23	23,065-24,636	523	Myristylated membrane protein	ORF_061L [Scale drop disease virus]	0	YP_009163822
24	24,675-26,084	469	Hypothetical protein	ORF_062R [Scale drop disease virus]	3.78E-93	YP_009163823
25	26,604-27,050	136	Hypothetical protein	ORF_063L [Scale drop disease virus]	6.39E-33	YP_009163824
26	27,351-27,665	104	RING E3 ubiquitin ligase	ORF_064R [Scale drop disease virus]	3.44E-37	YP_009163825
27	27,761-29,290	509	Serine/threonine protein kinase	ORF_065R [Scale drop disease virus]	5.56E-164	YP_009163826

Table S1. Continued.

ORF	Position (nt range)	Product size (AA)	Predicted function and conserved domain or signature	Best BLAST hit[a]		
				Description	E-value	Accession no.
28	29,352-29,678	108	Hypothetical protein	ORF_066L [Scale drop disease virus]	9.29E-36	YP_009163827
29	29,699-31,576	625	Macro domain-containing protein	ORF_067R [Scale drop disease virus]	0	YP_009163828
30	31,586-32,428	280	IKI3 family protein	ORF_068R [Scale drop disease virus]	4.28E-127	YP_009163829
31	32,611-33,624	337	Serpin (serine protease inhibitor)	ORF_097L [Scale drop disease virus]	2.92E-71	YP_009163858
32	33,711-34,373	220	Hypothetical protein	ORF_075R [Scale drop disease virus]	3.99E-118	YP_009163836
33	34,709-35,614	301	Hypothetical protein	062L [Cherax quadricarinatus iridovirus]	2.50E-13	ASZ85042
34	36,131-36,550	139	SAP domain-containing protein	ORF_027L [Scale drop disease virus]	2.10E-63	YP_009163788
35	36,571-37,101	176	Macro domain-containing protein	PREDICTED: O-acetyl-ADP-ribose deacetylase 1 [Nothobranchius furzeri]	2.67E-60	XP_015823954
36	37,175-38,845	556	Hypothetical protein	ORF_028R [Scale drop disease virus]	0	YP_009163789
37	38,894-39,520	208	Hypothetical protein	ORF_054R [Scale drop disease virus]	1.08E-11	YP_009163815
38	29,676-41,655	659	Hypothetical protein	ORF_128R [Scale drop disease virus]	6.66E-68	YP_009163889
39	41,661-43,334	557	Hypothetical protein	ORF_127R [Scale drop disease virus]	1.35E-134	YP_009163888
40	43,382-43,615	77	Hypothetical protein	ORF_126R [Scale drop disease virus]	2.40E-29	YP_009163887
41	44,385-45,743	452	Major capsid protein	ORF_060L [Scale drop disease virus]	0	YP_009163821

Table S1. Continued.

ORF	Position (nt range)	Product size (AA)	Predicted function and conserved domain or signature	Best BLAST hit[a]		
				Description	E-value	Accession no.
42	47,071-47,508	145	Hypothetical protein	ORF_110R [Scale drop disease virus]	2.10E-47	YP_009163871
43	47,581-47,940	119	Hypothetical protein	ORF_058R [Scale drop disease virus]	3.34E-27	YP_009163819
44	47,947-49,368	473	Ankyrin repeat-containing protein	ORF_108R [Scale drop disease virus]	8.73E-104	YP_009163869
45	49,385-49,918	177	Hypothetical protein	hypothetical protein [Pompano iridovirus]	5.22E-52	AZQ20780
46	49,964-51,400	478	Ankyrin repeat-containing protein	ORF_107L [Scale drop disease virus]	5.49E-70	YP_009163868
47	51,653-53,059	468	Ankyrin repeat-containing protein	ORF_116R [Scale drop disease virus]	4.45E-60	YP_009163877
48	53,570-54,124	184	Hypothetical protein	hypothetical protein LCDV1gp087 [Lymphocystis disease virus 1]	1.86E-04	NP_078741
49	54,277-54,615	112	HIRAN domain-containing protein	hypothetical protein K493DRAFT_308693 [Basidiobolus meristosporus CBS 931.73]	5.67E-09	ORX78499
50	54,662-55,636	324	Proteasome accessory factor (PafA2)	ORF_031L [Scale drop disease virus]	1.12E-97	YP_009163792
51	55,790-56,881	363	Hypothetical protein	ORF_030L [Scale drop disease virus]	2.12E-157	YP_009163791
52	56,884-57,417	177	Hypothetical protein	ORF_029L [Scale drop disease virus]	1.01E-57	YP_009163790
53	57,527-58,447	306	Hypothetical protein	ORF_055R [Scale drop disease virus]	6.15E-79	YP_009163816

Table S1. Continued.

ORF	Position (nt range)	Product size (AA)	Predicted function and conserved domain or signature	Best BLAST hit[a]		
				Description	E-value	Accession no.
54	58,582-59,580	332	Ribonucleotide reductase beta subunit	ribonucleoside-diphosphate reductase subunit M2-like [Oncorhynchus kisutch]	1.04E-177	XP_020351890
55	59,967-60,746	259	Deoxyribonuclease 1	deoxyribonuclease-1-like [Acanthochromis polyacanthus]	5.49E-95	XP_022075590
56	60,743-61,357	204	Hypothetical protein	ORF_086R [Scale drop disease virus]	5.22E-59	YP_009163847
57	62,124-63,071	315	Serpin (serine protease inhibitor)	ORF_045R [Scale drop disease virus]	5.69E-77	YP_009163806
58	63,574-64,605	343	Hypothetical protein			
59	64,984-70,611	1875	Hypothetical protein	ORF_105L [Scale drop disease virus]	0	YP_009163866
60	70,631-73,369	912	DEAD-like helicase	ORF_104L [Scale drop disease virus]	0	YP_009163865
61	73,898-74,617	239	RING E3 ubiquitin ligase	ORF_103L [Scale drop disease virus]	2.82E-10	YP_009163864
62	74,655-74,972	105	RING E3 ubiquitin ligase	ORF_102L [Scale drop disease virus]	4.26E-45	YP_009163863
63	74,891-75,322	143	Ankyrin repeat-containing protein	ORF_100L [Scale drop disease virus]	8.64E-40	YP_009163861
64	75,327-75,890	187	NIF/NLI interacting factor	ORF_099L [Scale drop disease virus]	7.30E-95	YP_009163860
65	76,300-77,688	462	Hypothetical protein			
66	77,695-79,245	516	Chromosome segregation protein			

Table S1. Continued.

ORF	Position (nt range)	Product size (AA)	Predicted function and conserved domain or signature	Best BLAST hit[a]		
				Description	E-value	Accession no.
67	79,473-80,582	369	Hypothetical protein	ORF_002R [Scale drop disease virus]	1.67E-53	YP_009163763
68	81,271-81,627	118	Ervl/Alr family protein	ORF_003L [Scale drop disease virus]	4.17E-66	YP_009163764
69	81,631-83,097	488	Hypothetical protein	ORF_004L [Scale drop disease virus]	3.54E-70	YP_009163765
70	83,111-84,664	517	Hypothetical protein	ORF_005L [Scale drop disease virus]	2.21E-59	YP_009163766
71	84,657-85,352	231	Cytosine DNA methyltransferase	cytosine DNA methyltransferase [Three spot gourami iridovirus]	1.77E-91	AVR29817
72	85,931-86,302	123	Hypothetical protein	ORF_010R [Scale drop disease virus]	1.25E-46	YP_009163771
73	86,299-86,694	131	Vascular endothelial growth factors	ORF_011R [Scale drop disease virus]	1.12E-14	YP_009163772
74	86,954-87,358	134	Bcl-2 family apoptosis regulator protein	ORF_013L [Scale drop disease virus]	1.69E-28	YP_009163774
75	87,622-88,560	312	2-cysteine adaptor domain-containing protein	ORF_014L [Scale drop disease virus]	9.82E-105	YP_009163775
76	88,572-89,417	281	2-cysteine adaptor domain-containing protein	ORF_015L [Scale drop disease virus]	4.28E-77	YP_009163776
77	89,426-90,061	211	Hypothetical protein	ORF_016L [Scale drop disease virus]	8.91E-118	YP_009163777
78	90,440-90,721	93	Hypothetical protein	hypothetical protein ORF 099R [Red seabream iridovirus]	2.27E-09	BAK14229
79	90,718-91,569	283	Transcription factor	ORF_017L [Scale drop disease virus]	5.21E-149	YP_009163778

Table S1. Continued.

ORF	Position (nt range)	Product size (AA)	Predicted function and conserved domain or signature	Best BLAST hit[a]		
				Description	E-value	Accession no.
80	91,580-92,371	263	Hypothetical protein	ORF_018L [Scale drop disease virus]	9.20E-91	YP_009163779
81	92,373-94,754	793	Hypothetical protein	ORF_018L [Scale drop disease virus]	1.07E-129	YP_009163779
82	95,308-95,658	116	Hypothetical protein			
83	95,663-96,217	184	Hypothetical protein	ORF_059R [Scale drop disease virus]	9.16E-13	YP_009163820
84	96,473-96,709	78	Ubiquitin family protein	40S ribosomal protein S27a [Cyberlindnera jadinii]	2.47E-41	CEP23256
85	97,430-100,306	988	Hypothetical protein	ORF_091L [Scale drop disease virus]	0	YP_009163852
86	100,334-100,831	165	Hypothetical protein	ORF_092R [Scale drop disease virus]	1.04E-51	YP_009163853
87	100,837-101,337	166	Hypothetical protein	ORF_093R [Scale drop disease virus]	8.74E-47	YP_009163854
88	101,374-102,555	393	Hypothetical protein	ORF_094L [Scale drop disease virus]	8.14E-150	YP_009163855
89	102,845-103,927	354	Hypothetical protein			
90	104,437-107,211	924	D5 family NTPase	ORF_040R [Scale drop disease virus]	0	YP_009163801
91	107,140-107,964	274	Proliferating cell nuclear antigen	ORF_039L [Scale drop disease virus]	6.23E-161	YP_009163800
92	108,026-109,318	430	Ankyrin repeat-containing protein	ORF_115R [Scale drop disease virus]	2.45E-28	YP_009163876
93	109,488-110,072	194	Hypothetical protein	ORF_038R [Scale drop disease virus]	1.47E-103	YP_009163799

Table S1. Continued.

ORF	Position (nt range)	Product size (AA)	Predicted function and conserved domain or signature	Best BLAST hit[a]		
				Description	E-value	Accession no.
94	110,026-112,656	876	Tyrosine kinase	ORF_O37R [Scale drop disease virus]	0	YP_009163798
95	112,747-113,784	345	Immediate early protein ICP-46	ORF_036L [Scale drop disease virus]	1.68E-153	YP_009163797
96	113,790-114,512	240	ATPase	ORF_035L [Scale drop disease virus]	1.83E-158	YP_009163796
97	114,614-115,201	195	US22 protein	uncharacterized protein LOC109953611 isoform X1 [Monopterus albus]	1.38E-24	XP_020444743
98	115,441-116,145	234	Hypothetical protein	ORF_034R [Scale drop disease virus]	2.06E-122	YP_009163795
99	116,199-117,629	476	Ankyrin repeat-containing protein	ORF_116R [Scale drop disease virus]	3.35E-51	YP_009163877
100	117,681-118,499	272	Hypothetical protein	ORF_033L [Scale drop disease virus]	3.48E-103	YP_009163794
101	118,506-119,699	397	Hypothetical protein	ORF_032L [Scale drop disease virus]	2.41E-119	YP_009163793
102	119,855-121,282	475	Ankyrin repeat-containing protein	ORF_116R [Scale drop disease virus]	1.34E-70	YP_009163877
103	121,342-122,253	303	Flap endonuclease	ORF_073L [Scale drop disease virus]	9.10E-164	YP_009163834
104	122,276-125,833	1185	DNA-dependent RNA polymerase II largest subunit	ORF_076L [Scale drop disease virus]	0	YP_009163837
105	125,823-126,683	286	Hypothetical protein	ORF_078L [Scale drop disease virus]	1.22E-71	YP_009163839
106	126,705-127,223	172	Hypothetical protein	ORF_079R [Scale drop disease virus]	2.48E-87	YP_009163840

Table S1. Continued.

ORF	Position (nt range)	Product size (AA)	Predicted function and conserved domain or signature	Best BLAST hit[a]		
				Description	E-value	Accession no.
107	127,232-127,735	167	Hypothetical protein	ORF_080R [Scale drop disease virus]	7.32E-62	YP_009163841
108	127,722-128,210	162	Hypothetical protein	ORF_081R [Scale drop disease virus]	5.93E-50	YP_009163842

[a]Significant hits based on NCBI BLASTP analyses

Abbreviations: nt, nucleotides; AA, amino acids

Table S2. Genetic relationship among iridoviruses measured as nucleotide sequence identity in the major capsid protein. Values for European chub iridovirus are outlined in red. See Table 1 for virus abbreviations.

	LCDV1	LCDVC	LCDVSa	AMIV	IIV22	IIV22a	IIV30	IIV9	IIV25	IIV3	IIV6	IIV31	GIV	SGIV	ECV	ESV	SERV	CoIV	Rmax	ATV	EHNV	CH8_96	ADRV	CMTVM	PPIV	CMTVVB	FV3	SSME	RGV	STIV	TFV	TORV	GGRV	BIV	OSGIV	RSIV_RIE1	GSIVK1	RBIV-C1	RBIV	RSIV	RSIVKu	ISKNV	SACIV	TSGIV	TRBIV	SDDV	ECIV
LCDV1	-
LCDVC	87.6	-
LCDVSa	87.6	95.2	-
AMIV	52.1	53.0	52.3	-
IIV22	52.6	53.3	52.6	82.3	-
IIV22a	52.6	53.3	52.6	82.3	100.0	-
IIV30	52.4	53.1	52.9	82.3	99.6	99.6	-
IIV9	52.2	53.1	52.7	82.0	95.4	95.4	95.0	-
IIV25	52.1	53.0	52.5	82.1	96.1	96.1	95.6	97.7	-
IIV3	52.7	53.1	52.4	72.5	74.6	74.6	74.4	74.7	74.6	-
IIV6	55.0	53.9	54.4	71.6	71.5	71.5	71.1	69.7	69.8	62.9	-
IIV31	53.0	51.6	52.1	71.4	68.3	68.3	68.1	67.8	68.0	61.6	86.6	-
GIV	54.0	54.8	53.9	45.1	45.8	45.8	45.6	44.9	45.0	43.1	47.0	46.1	-
SGIV	54.0	54.8	53.9	45.1	45.8	45.8	45.6	44.9	45.0	43.1	47.0	46.1	99.8	-
ECV	52.8	52.0	51.7	45.3	47.4	47.4	48.1	45.5	45.6	45.1	47.0	47.2	73.0	73.0	-
ESV	52.8	52.0	51.7	45.3	47.4	47.4	48.1	45.5	45.6	45.1	47.0	47.2	73.0	73.0	100.0	-
SERV	52.4	52.0	51.7	45.3	47.1	47.1	47.1	45.5	45.6	44.7	46.5	47.2	73.0	73.0	97.0	97.0	-
CoIV	52.6	51.7	51.7	45.5	46.4	46.4	47.1	44.4	44.5	44.5	46.3	46.7	72.6	72.6	97.0	97.0	95.2	-
Rmax	52.6	51.7	51.7	45.5	46.4	46.4	47.1	44.4	44.5	44.5	46.3	46.7	72.6	72.6	97.0	97.0	95.2	100.0	-
ATV	52.8	51.5	51.5	45.3	46.3	46.3	47.0	44.4	44.5	44.0	46.1	46.3	72.4	72.4	97.0	97.0	94.8	97.0	97.0	-
EHNV	52.6	51.4	51.2	44.8	45.9	45.9	45.9	44.9	45.0	44.2	46.3	46.7	72.1	72.1	97.8	97.8	96.1	97.2	97.2	98.1	-
CH8_96	52.6	51.5	51.3	45.3	46.4	46.4	47.1	45.3	45.4	44.7	46.5	46.9	72.8	72.8	97.6	97.6	95.9	97.4	97.4	97.8	98.9	-
ADRV	52.6	51.5	51.3	45.3	46.4	46.4	47.1	45.3	45.4	44.7	46.5	46.9	72.8	72.8	97.6	97.6	95.9	97.4	97.4	97.8	98.9	100.0	-
CMTVM	52.6	51.5	51.3	45.3	46.4	46.4	47.1	45.3	45.4	44.7	46.5	46.9	73.0	73.0	97.4	97.4	95.7	97.2	97.2	97.6	98.7	99.8	99.8	-
PPIV	52.6	51.5	51.3	45.5	46.2	46.2	46.9	45.5	45.6	44.5	46.3	46.7	73.2	73.2	97.2	97.2	95.5	97.0	97.0	97.4	98.5	99.6	99.6	99.8	-
CMTVVB	52.8	51.7	51.5	45.3	46.4	46.4	47.1	45.3	45.4	44.5	46.5	46.9	73.2	73.2	97.6	97.6	95.9	97.4	97.4	97.4	98.5	99.6	99.6	99.8	99.6	-
FV3	52.2	51.3	51.1	45.5	46.4	46.4	47.1	45.5	45.6	44.7	46.9	46.9	72.8	72.8	95.9	95.9	95.0	95.7	95.7	95.7	96.8	97.8	97.8	98.1	97.8	98.3	-
SSME	52.2	51.3	51.1	45.5	46.4	46.4	47.1	45.5	45.6	44.7	47.1	46.9	73.0	73.0	96.1	96.1	95.2	95.9	95.9	95.9	97.0	98.1	98.1	98.3	98.1	98.5	99.8	-
RGV	52.2	51.5	51.1	45.5	46.4	46.4	47.1	45.3	45.4	44.7	47.1	46.9	73.2	73.2	96.3	96.3	95.5	96.1	96.1	96.1	97.2	98.3	98.3	98.5	98.3	98.7	99.6	99.8	-
STIV	52.2	51.5	51.1	45.5	46.4	46.4	47.1	45.3	45.4	44.7	47.1	46.9	73.2	73.2	96.3	96.3	95.5	96.1	96.1	96.1	97.2	98.3	98.3	98.5	98.3	98.7	99.6	99.8	100.0	-
TFV	52.2	51.5	51.1	45.5	47.2	47.2	47.2	45.5	45.6	44.9	47.1	46.9	72.8	72.8	95.7	95.7	95.2	95.7	95.7	96.1	96.8	97.8	97.8	98.1	97.8	97.8	98.3	98.5	98.7	98.7	-
TORV	52.2	51.5	51.1	45.5	46.6	46.6	47.4	45.5	45.6	44.9	47.3	47.2	73.2	73.2	96.1	96.1	95.2	95.9	95.9	96.3	97.4	98.5	98.5	98.7	98.5	98.5	98.9	99.1	99.4	99.4	98.9	-
GGRV	52.0	51.3	50.9	45.5	46.6	46.6	47.4	45.5	45.6	44.9	47.1	46.9	73.0	73.0	96.3	96.3	95.5	96.1	96.1	96.5	97.6	98.7	98.7	98.9	98.7	98.7	99.1	99.4	99.6	99.6	99.1	99.8	-
BIV	52.0	51.3	50.9	45.5	46.6	46.6	47.4	45.5	45.6	44.9	47.1	46.9	73.0	73.0	96.3	96.3	95.5	96.1	96.1	96.5	97.6	98.7	98.7	98.9	98.7	98.7	99.1	99.4	99.6	99.6	99.1	99.8	100.0	-
OSGIV	50.0	49.1	49.6	46.0	46.4	46.4	46.4	45.5	45.4	46.0	47.8	46.4	45.8	45.8	47.8	47.8	47.8	48.0	48.0	48.0	47.8	48.0	48.0	48.0	48.2	48.2	48.0	48.0	48.0	48.0	48.2	47.8	47.8	47.8	-
RSIV_RIE12-1	50.0	49.1	49.6	46.0	46.4	46.4	46.4	45.5	45.4	46.0	47.8	46.4	45.8	45.8	47.8	47.8	47.8	48.0	48.0	48.0	47.8	48.0	48.0	48.0	48.2	48.2	48.0	48.0	48.0	48.0	48.2	47.8	47.8	47.8	100.0	-
GSIVK1	50.0	49.1	49.6	46.0	46.4	46.4	46.4	45.5	45.4	46.0	47.8	46.4	45.8	45.8	47.8	47.8	47.8	48.0	48.0	48.0	47.8	48.0	48.0	48.0	48.2	48.2	48.0	48.0	48.0	48.0	48.2	47.8	47.8	47.8	100.0	100.0	-
RBIV-C1	50.0	49.1	49.6	46.0	46.4	46.4	46.4	45.5	45.4	46.0	47.8	46.4	45.8	45.8	47.8	47.8	47.8	48.0	48.0	48.0	47.8	48.0	48.0	48.0	48.2	48.2	48.0	48.0	48.0	48.0	48.2	47.8	47.8	47.8	100.0	100.0	100.0	-
RBIV	49.8	48.9	49.3	45.8	46.2	46.2	46.2	45.2	45.2	45.8	47.6	46.2	45.8	45.8	47.8	47.8	47.8	48.0	48.0	48.0	47.8	48.0	48.0	48.0	48.2	48.2	48.0	48.0	48.0	48.0	48.2	47.8	47.8	47.8	99.6	99.6	99.6	99.6	-
RSIV	50.0	49.1	49.6	46.0	46.6	46.6	46.6	45.5	45.4	45.8	47.8	46.4	45.6	45.6	47.8	47.8	47.8	48.0	48.0	48.0	47.8	48.0	48.0	48.0	48.2	48.2	48.0	48.0	48.0	48.0	48.2	47.8	47.8	47.8	99.6	99.6	99.6	99.6	99.1	-
RSIVKu	50.2	49.6	50.0	46.0	46.4	46.4	46.4	46.5	46.5	46.8	47.8	46.7	46.2	46.2	48.0	48.0	48.0	48.5	48.5	48.2	48.0	48.2	48.2	48.2	48.5	48.5	48.2	48.2	48.2	48.2	48.5	48.0	48.0	48.0	98.7	98.7	98.7	98.7	98.2	98.2	-
ISKNV	50.2	49.6	50.0	46.0	46.4	46.4	46.4	46.5	46.5	46.8	47.8	46.7	46.2	46.2	48.0	48.0	48.0	48.5	48.5	48.2	48.0	48.2	48.2	48.2	48.5	48.5	48.2	48.2	48.2	48.2	48.5	48.0	48.0	48.0	98.7	98.7	98.7	98.7	98.2	98.2	100.0	-
SACIV	49.8	48.9	49.3	45.8	46.8	46.8	46.8	46.0	46.3	46.6	47.3	46.4	46.0	46.0	47.3	47.3	47.3	47.8	47.8	47.8	47.3	47.6	47.6	47.6	47.8	47.8	47.6	47.6	47.6	47.6	47.8	47.3	47.3	47.3	97.6	97.6	97.6	97.6	97.1	97.4	98.0	98.0	-
TSGIV	49.8	48.9	49.3	45.8	46.8	46.8	46.8	46.0	46.3	46.6	47.3	46.4	46.0	46.0	47.3	47.3	47.3	47.8	47.8	47.8	47.3	47.6	47.6	47.6	47.8	47.8	47.6	47.6	47.6	47.6	47.8	47.3	47.3	47.3	97.6	97.6	97.6	97.6	97.1	97.4	98.0	98.0	100.0	-
TRBIV	49.8	48.9	49.3	46.0	47.0	47.0	47.0	46.2	46.2	46.0	47.6	46.4	45.6	45.6	47.3	47.3	47.3	47.8	47.8	47.8	47.3	47.6	47.6	47.6	47.8	47.8	47.6	47.6	47.6	47.6	47.8	47.3	47.3	47.3	98.0	98.0	98.0	98.0	97.6	97.6	98.0	98.0	98.7	98.7	-
SDDV	49.2	52.3	51.9	46.6	46.3	46.3	46.3	45.5	45.2	46.9	47.7	45.9	46.5	46.5	48.0	48.0	48.2	47.8	47.8	47.3	48.2	48.2	48.2	47.8	48.0	48.0	48.0	48.0	48.0	48.0	48.0	47.8	47.8	47.8	69.6	69.6	69.6	69.6	69.4	69.8	69.2	69.2	69.0	69.0	69.0	-
**ECIV**	**49.3**	**52.2**	**51.8**	**46.6**	**47.7**	**47.7**	**47.2**	**47.2**	**47.0**	**46.5**	**47.0**	**46.2**	**47.1**	**47.1**	**45.1**	**45.1**	**46.2**	**45.4**	**45.4**	**45.1**	**45.8**	**45.6**	**45.6**	**45.4**	**45.6**	**45.6**	**45.8**	**45.8**	**45.8**	**45.8**	**45.6**	**45.4**	**45.4**	**45.4**	**65.7**	**65.7**	**65.7**	**65.7**	**65.5**	**65.9**	**65.7**	**65.7**	**65.3**	**65.3**	**65.5**	**81.6**	-

Table S3. Genetic relationship among iridoviruses measured as nucleotide sequence identity in the ATPase gene. Values for European chub iridovirus are outlined in red. See Table 1 for virus abbreviations.

	GIV	SGIV	ATV	ECV	ESV	SERV	EHNV	CoIV	Rmax	CMTVM	TORV	FV3	SSME	RGV	STIV	GGRV	BIV	TFV	ADRV	CH8_96	CMTVVB	PPIV	RBIV-C1	OSGIV	RSIV_RIE12-1	GSIVK1	RSIV	RBIV	SACIV	TSGIV	TRBIV	RSIVKu	ISKNV	SDDV	ECIV	LCDV1	LCDVC	LCDVSa	AMIV	IIV22	IIV22a	IIV30	IIV9	IIV25	IIV3	IIV6	IIV31
GIV	-
SGIV	98.5	-
ATV	70.7	71.0	-
ECV	68.8	69.1	98.1	-
ESV	68.8	69.1	98.1	100.0	-
SERV	70.0	70.4	97.7	99.0	99.0	-
EHNV	70.0	70.4	98.4	99.0	99.0	98.7	-
CoIV	69.1	69.4	98.1	98.1	98.1	98.1	98.4	-
Rmax	69.1	69.4	98.1	98.1	98.1	98.1	98.4	100.0	-
CMTVM	69.4	69.7	98.4	98.1	98.1	98.4	98.7	98.1	98.1	-
TORV	69.7	70.1	98.1	98.1	98.1	98.1	98.4	98.1	98.1	99.4	-
FV3	69.7	70.1	97.7	98.1	98.1	97.7	98.1	97.8	97.8	99.4	99.0	-
SSME	69.4	69.7	97.4	97.8	97.8	97.4	97.7	97.5	97.5	99.0	98.7	99.7	-
RGV	69.7	70.1	98.1	97.8	97.8	97.7	98.4	97.8	97.8	99.0	99.0	99.4	99.0	-
STIV	69.7	70.1	98.1	97.8	97.8	97.7	98.4	97.8	97.8	99.0	99.0	99.4	99.0	100.0	-
GGRV	69.7	70.1	97.4	97.1	97.1	97.1	97.7	97.1	97.1	98.4	98.4	98.7	98.4	99.0	99.0	-
BIV	69.7	70.1	97.1	97.1	97.1	97.1	97.4	97.1	97.1	98.4	98.4	98.7	98.4	98.7	98.7	99.4	-
TFV	70.1	70.4	97.4	97.1	97.1	97.1	97.4	97.1	97.1	98.4	98.4	98.7	98.4	98.7	98.7	98.4	98.4	-
ADRV	69.4	69.7	97.7	97.8	97.8	97.7	98.1	97.8	97.8	99.0	99.0	99.4	99.0	99.4	99.4	98.7	98.7	98.7	-
CH8_96	69.7	70.1	98.1	98.1	98.1	98.1	98.4	98.1	98.1	99.4	99.4	99.7	99.4	99.7	99.7	99.0	99.0	99.0	99.7	-
CMTVVB	69.7	70.1	98.1	98.1	98.1	98.1	98.4	98.1	98.1	99.4	99.4	99.7	99.4	99.7	99.7	99.0	99.0	99.0	99.7	100.0	-
PPIV	69.7	70.1	98.1	98.1	98.1	98.1	98.4	98.1	98.1	99.4	99.4	99.7	99.4	99.7	99.7	99.0	99.0	99.0	99.7	100.0	100.0	-
RBIV-C1	54.2	54.6	51.9	51.9	51.9	51.9	51.9	51.5	51.5	52.3	52.3	52.3	52.3	52.3	52.3	52.3	52.3	51.5	52.3	52.3	52.3	52.3	-
OSGIV	54.2	54.6	51.9	51.9	51.9	51.9	51.9	51.5	51.5	52.3	52.3	52.3	52.3	52.3	52.3	52.3	52.3	51.5	52.3	52.3	52.3	52.3	100.0	-
RSIV_RIE12-1	54.2	54.6	51.9	51.9	51.9	51.9	51.9	51.5	51.5	52.3	52.3	52.3	52.3	52.3	52.3	52.3	52.3	51.5	52.3	52.3	52.3	52.3	100.0	100.0	-
GSIVK1	54.2	54.6	51.9	51.9	51.9	51.9	51.9	51.5	51.5	52.3	52.3	52.3	52.3	52.3	52.3	52.3	52.3	51.5	52.3	52.3	52.3	52.3	100.0	100.0	100.0	-
RSIV	54.2	54.6	51.9	51.9	51.9	51.9	51.9	51.5	51.5	52.3	52.3	52.3	52.3	52.3	52.3	52.3	52.3	51.5	52.3	52.3	52.3	52.3	100.0	100.0	100.0	100.0	-
RBIV	53.8	54.2	51.5	51.5	51.5	51.5	51.5	51.0	51.0	51.9	51.9	51.9	51.9	51.9	51.9	51.9	51.9	51.0	51.9	51.9	51.9	51.9	99.6	99.6	99.6	99.6	99.6	-
SACIV	54.2	54.6	51.5	51.5	51.5	51.5	51.5	51.0	51.0	51.9	51.9	51.9	51.9	51.9	51.9	51.9	51.9	51.0	51.9	51.9	51.9	51.9	99.6	99.6	99.6	99.6	99.6	99.2	-
TSGIV	54.2	54.6	51.5	51.5	51.5	51.5	51.5	51.0	51.0	51.9	51.9	51.9	51.9	51.9	51.9	51.9	51.9	51.0	51.9	51.9	51.9	51.9	99.6	99.6	99.6	99.6	99.6	99.2	100.0	-
TRBIV	53.8	54.2	52.3	52.3	52.3	52.3	52.3	51.9	51.9	52.7	52.7	52.7	52.7	52.7	52.7	52.7	52.7	51.9	52.7	52.7	52.7	52.7	98.7	98.7	98.7	98.7	98.7	98.3	98.3	98.3	-
RSIVKu	54.2	54.2	53.1	53.1	53.1	53.1	52.7	52.7	52.7	53.1	53.1	53.1	53.6	53.1	53.1	53.1	53.1	52.7	53.1	53.1	53.1	53.1	98.7	98.7	98.7	98.7	98.7	98.3	98.3	98.3	98.3	-
ISKNV	54.2	54.2	53.1	53.1	53.1	53.1	52.7	52.7	52.7	53.1	53.1	53.1	53.6	53.1	53.1	53.1	53.1	52.7	53.1	53.1	53.1	53.1	98.7	98.7	98.7	98.7	98.7	98.3	98.3	98.3	98.3	100.0	-
SDDV	54.9	54.5	53.1	52.2	52.2	52.2	52.2	52.2	52.2	52.2	52.2	52.2	52.2	52.2	52.2	52.2	51.8	52.2	52.2	52.2	52.2	52.2	70.7	70.7	70.7	70.7	70.7	70.3	70.7	70.7	70.3	72.4	72.4	-
**ECIV**	**54.8**	**55.6**	**53.3**	**53.8**	**53.8**	**53.8**	**53.8**	**53.8**	**53.8**	**53.8**	**53.8**	**53.8**	**53.8**	**53.8**	**53.8**	**53.8**	**54.2**	**53.3**	**53.8**	**53.8**	**53.8**	**53.8**	**71.0**	**71.0**	**71.0**	**71.0**	**71.0**	**70.6**	**70.6**	**70.6**	**70.6**	**71.8**	**71.8**	**88.3**	-
LCDV1	58.8	58.8	53.9	53.5	53.5	53.5	53.5	53.9	53.9	53.5	53.5	53.5	53.5	53.5	53.5	53.5	53.5	53.5	53.5	53.5	53.5	53.5	55.5	55.5	55.5	55.5	55.5	55.0	55.5	55.5	55.5	55.5	55.5	57.9	**57.0**	-
LCDVC	58.8	58.8	56.0	56.0	56.0	56.0	56.0	56.0	56.0	56.0	56.0	56.0	56.0	56.0	56.0	56.0	56.0	56.0	56.0	56.0	56.0	56.0	55.0	55.0	55.0	55.0	55.0	54.6	55.0	55.0	55.0	55.9	55.9	58.3	**56.1**	81.6	-
LCDVSa	58.4	58.4	57.2	57.2	57.2	57.2	57.2	57.2	57.2	57.2	57.2	57.2	57.2	57.2	57.2	57.2	57.2	57.2	57.2	57.2	57.2	57.2	55.9	55.9	55.9	55.9	55.9	55.5	55.9	55.9	55.9	56.7	56.7	57.9	**56.9**	82.0	94.7	-
AMIV	39.7	39.7	41.3	41.0	41.0	41.0	41.8	41.8	41.8	41.8	41.8	41.8	41.8	41.8	41.8	41.8	41.8	41.8	42.6	41.8	41.8	41.8	38.1	38.1	38.1	38.1	38.1	38.1	37.7	37.7	38.6	39.0	39.0	41.7	**40.5**	44.8	46.5	45.2	-
IIV22	40.7	41.1	42.0	42.0	42.0	42.0	42.0	42.0	42.0	42.0	42.0	42.0	42.0	42.0	42.0	42.0	42.0	42.0	42.0	42.0	42.0	42.0	39.0	39.0	39.0	39.0	39.0	39.0	38.6	38.6	39.4	39.4	39.4	41.7	**42.1**	44.6	45.4	44.2	84.7	-
IIV22a	40.6	41.0	42.0	42.0	42.0	42.0	42.0	42.0	42.0	42.0	42.0	42.0	42.0	42.0	42.0	42.0	42.0	42.0	42.0	42.0	42.0	42.0	39.0	39.0	39.0	39.0	39.0	39.0	38.6	38.6	39.4	39.4	39.4	41.7	**42.1**	44.6	45.4	44.2	84.7	100.0	-
IIV30	40.7	41.1	41.2	41.2	41.2	41.2	41.2	41.2	41.2	41.2	41.2	41.2	41.2	41.2	41.2	41.2	41.2	41.2	41.2	41.2	41.2	41.2	39.0	39.0	39.0	39.0	39.0	39.0	38.6	38.6	39.4	39.4	39.4	42.6	**42.9**	44.6	45.4	43.3	85.5	99.2	99.2	-
IIV9	39.8	40.2	41.2	41.2	41.2	41.2	41.2	41.2	41.2	41.2	41.2	41.6	41.6	41.2	41.2	41.2	41.2	41.2	41.2	41.2	41.2	41.2	39.8	39.8	39.8	39.8	39.8	39.8	39.4	39.4	40.3	39.4	39.4	41.7	**41.4**	44.0	44.8	43.2	83.5	92.8	92.9	93.6	-
IIV25	39.8	40.2	41.2	41.2	41.2	41.2	41.2	41.2	41.2	41.2	41.2	41.6	41.6	41.2	41.2	41.2	41.2	41.2	41.2	41.2	41.2	41.2	39.0	39.0	39.0	39.0	39.0	39.0	38.6	38.6	39.4	39.4	39.4	41.7	**41.4**	44.0	44.8	43.2	82.7	91.2	91.3	92.0	98.4	-
IIV3	39.4	39.4	38.7	38.7	38.7	38.7	38.7	38.7	38.7	39.1	39.1	39.1	39.1	39.1	39.1	39.1	39.1	39.1	39.1	39.1	39.1	39.1	38.2	38.2	38.2	38.2	38.2	38.2	37.8	37.8	38.2	39.1	39.1	38.5	**40.9**	45.0	45.5	44.2	75.1	76.7	77.4	77.1	77.8	76.6	-
IIV6	42.0	41.6	38.1	38.1	38.1	38.1	38.1	38.1	38.1	38.9	38.9	38.9	38.9	38.9	38.9	38.9	38.9	38.9	38.1	38.9	38.9	38.9	38.9	38.9	38.9	38.9	38.9	38.5	38.5	38.5	39.7	39.3	39.3	41.2	**40.8**	40.7	41.6	40.7	51.6	50.2	50.0	50.6	49.6	49.6	47.2	-
IIV31	39.3	40.1	37.9	37.9	37.9	37.9	37.9	39.1	39.1	37.9	37.9	38.7	38.7	37.9	37.9	37.9	37.8	37.9	37.9	37.9	37.9	37.9	37.2	37.2	37.2	37.2	37.2	36.8	36.8	36.8	38.1	37.2	37.2	39.0	**39.2**	40.7	40.3	39.7	50.6	49.4	49.2	50.2	50.4	50.0	46.1	82.6	-
